# Effect of oral intake of capsaicinoid beadlets on catecholamine secretion and blood markers of lipolysis in healthy adults: a randomized, placebo controlled, double-blind, cross-over study

**DOI:** 10.1186/1476-511X-9-72

**Published:** 2010-07-15

**Authors:** Richard J Bloomer, Robert E Canale, Sid Shastri, Sujata Suvarnapathki

**Affiliations:** 1 Cardiorespiratory/Metabolic Laboratory, Department of Health and Sport Sciences, University of Memphis, Memphis, TN, USA; 2 Shastri Tech, Los Angeles, CA, USA; 3 Sankhya Analytical Research, Mumbai, India

## Abstract

**Background:**

In the present investigation we compared blood epinephrine (EPI), norepinephrine (NE), free fatty acids (FFA) and glycerol concentrations in response to a capsaicinoid supplement or placebo in healthy adults before and after acute exercise.

**Methods:**

Twenty subjects ingested a placebo or supplement (Capsimax™, OmniActive Health Technologies; 2 mg capsaicinoids in a microencapsulated matrix) with one week separating conditions. Fasting blood samples were collected during each visit; 30 minutes following a rest period and before placebo or supplement intake (Pre); 2 hours post intake (2 hr); one minute following the cessation of 30 minutes of exercise performed at 65% of maximal heart rate reserve (2.5 hr); 90 minutes following the cessation of exercise (4 hr). Heart rate (HR), systolic (SBP) and diastolic (DBP) blood pressure were recorded at all times.

**Results:**

A time effect was noted for HR, SBP, and DBP (p < 0.05), with HR and SBP higher at 2.5 hr compared to Pre (due to exercise) and DBP lower at 2.5 hr compared to Pre. No interaction or condition effects were noted for EPI, NE, FFA, or glycerol (p > 0.05). However, a time effect was noted for all variables (p < 0.0001), with values higher than Pre at 2.5 hr for EPI and glycerol, at 2 hr and 2.5 hours for FFA, and at 2 hr, 2.5 hr, and 4 hr for NE (p < 0.05). In terms of percent change from Pre, glycerol was higher with Capsimax™ than for placebo at 4 hr (p = 0.011) and FFA was higher with Capsimax™ than for placebo at 2 hr (p = 0.025) and at 2.5 hr (p = 0.015).

**Conclusion:**

Ingestion of low dose (2 mg) Capsimax™ was associated with an increase in blood FFA and glycerol at selected times post ingestion, as compared to placebo. However, Capsimax™ had no differing effect on EPI or NE compared to placebo. Lastly, no difference was noted in HR, SBP, or DBP between placebo and Capsimax™.

## Background

The prevalence of obesity and overweight status is clearly on the rise throughout the developed world, evidenced by the nearly 400 million individuals currently classified as obese [1,2], with an additional 1.6 billion classified as overweight [2]. While lifestyle factors such as increased physical activity [3] and modification of dietary intake [4] are viewed as a first line method in both the prevention and condition of obesity and obesity related illness, pharmaceuticals [5] and dietary supplements [6] are often used as an aid in body fat/weight loss. In relation to the latter, many such supplements have little to no scientific support in human subjects, while some have been reported to cause ill-health [7]. This is especially true in regards to the hemodynamic response observed with many dietary supplements targeting weight/fat loss [8], a real concern in particular for those who are hypertensive.

One nutritional agent with scientific support as an aid to weight/fat loss is capsaicinoids. Capsaicinoids are the major pungent, naturally occurring active compounds in capsicum fruits such as hot chili peppers (genus capsicum), with the most abundant forms being capsaicin (8-methyl-N-vanillyl-6-nonenamide), dihydrocapsaicin, and nordihydrocapsaicin [9]. Approximately 3 mg of capsaicinoids are present within 1 g of dried red pepper [10]. Capsaicinoids have been reported in the literature to reduce *ad libitum *food intake [11-13], to increase thermogenesis [10,14-16], and to increase lipolysis as measured via breath sample analysis [10,14,15,17]. Moreover, epidemiological data describe an association between the consumption of capsaicinoid containing foods and a lower incidence of obesity [18]. This appears to occur without any significant and prolonged effect on heart rate or blood pressure [19].

In terms of lipolysis, capsaicinoids are an agonist of the transient receptor potential vanilloid subfamily member 1 (TRPV1). This TRPV1 releases the substance P, which in turn activates the postsynaptic receptor of substance P, neurokinin-1. This activation of neurokin-1 results in an increased activation of the sympathetic nervous system, leading to the release of epinephrine (EPI) and norepinephrine (NE) from adrenal gland [20]. Because both EPI and NE interact with hormone sensitive lipase, an increase in triglyceride degradation may be observed with capsaicinoid intake, which may lead to an increase in circulating free fatty acids (FFA) and glycerol [21].

While many authors have suggested an increase in lipolysis following capsaicinoid intake [14,15], these studies have relied on the measurement of breath sample analysis (e.g., respiratory exchange ratio) as a crude measure of substrate utilization. To our knowledge, no studies using human subjects have determined fat oxidation by including the measurements of blood FFA and glycerol concentrations following capsaicinoid intake. Moreover, no human studies have included the measurements of blood EPI and NE concentrations following capsaicinoid intake. Finally, due to the fact that many individuals use nutritional supplements in conjunction with aerobic exercise for fat loss purposes, we were also interested in the combined effects of capsaicinoid treatment and exercise on markers of lipolysis. Therefore, the purpose of the present investigation was to compare blood EPI, NE, FFA, and glycerol concentrations before and at selected times through 4 hours post ingestion of a placebo or a capsaicinoid supplement, with the addition of an acute exercise bout. We hypothesized that the capsaicinoid supplement would result in an increase in circulating catecholamines and in markers of lipolysis compared to placebo.

## Methods

### Subjects

Young and healthy, exercise-trained men (n = 10) and women (n = 10) participated in this investigation. All subjects completed a detailed medical history and physical activity questionnaire in order to determine eligibility. No subject was a smoker, used smokeless tobacco products, or had diagnosed cardiovascular or metabolic disease. Men and women were considered to be exercise-trained, as they performed combined aerobic and anaerobic exercise for 7 ± 2 and 6 ± 2 hrs per week, respectively, for the past several years. Subject descriptive characteristics are presented in Table 1. All experimental procedures were performed in accordance with the Helsinki Declaration. The University of Memphis Human Subjects Committee approved all experimental procedures, and subjects provided verbal and written consent prior to participating in this study.

**Table 1:** Descriptive characteristics of 20 healthy adults

Variable	Men (n = 10)	Women (n = 10)	P value
Age (yrs)	25.5 ± 1.2	23.9 ± 1.3	0.375
Height (cm)	175.2 ± 2.0	165.9 ± 1.4	0.002
Weight (kg)	77.4 ± 2.5	60.2 ± 3.9	0.002
BMI (kg·m^-2^)	25.2 ± 0.6	21.7 ± 1.1	0.01
Body fat (%)*	10.2 ± 1.1	21.1 ± 1.6	< 0.0001
Waist (cm)	82.9 ± 1.7	67.5 ± 1.9	< 0.0001
Hip (cm)	99.0 ± 1.4	96.4 ± 2.8	0.428
Waist:Hip	0.84 ± 0.01	0.70 ± 0.01	< 0.0001
Anaerobic Exercise (years)	8.3 ± 1.5	4.2 ± 1.0	0.035
Anaerobic Exercise (hours per week)	3.6 ± 0.5	1.4 ± 0.4	0.002
Aerobic Exercise (years)	5.1 ± 1.4	7.9 ± 2.2	0.282
Aerobic Exercise (hours per week)	3.8 ± 1.2	5.0 ± 1.1	0.484

Data are Mean ± SEM

* Determined from 7-site skinfold analysis use Lange calipers and Siri equation

### Conditions and Testing

All procedures described below were identical for both test days (supplement and placebo). The dietary supplement used in this investigation (Capsimax™; OmniActive Health Technologies, Mumbai, India) included 100 mg of encapsulated beadlets, standardized to 2% capsaicinoids, of which 1.2-1.35% was capsaicin, 0.6-0.8% was dihydrocapsaicin, and 0.1-0.2% was nordihydrocapsaicin. While capsaicinoids are absorbed through the gastrointestinal tract by a non-active process and then distributed to the whole body via plasma [22], the supplement used in the present investigation is coated to minimize pungency and to avoid gastric irritation. It is specifically designed for minimal release in stomach (up to a maximum of 15% of capsaicinoids), but rather a release in the small intestine. Dissolution studies performed by OmniActive Health Technologies indicate a range of 60-75% of capsaicinoids released by 4 hours post ingestion of the supplement, when consumed on an empty stomach following an overnight fast. For complete release of capsaicinoids, the time of study would need to be extended beyond 6 hours. While such a timeframe may be ideal and provide for the most robust effects, we did not attempt this within the current design due to the difficulty in having subjects fast for such an extended period. Hence, the present study only carried out measures through 4 hours post ingestion.

The placebo capsules were identical in appearance and consisted of cellulose. Both the supplement and placebo capsules were manufactured under GMP conditions (Rubicon Research, Mumbai, India). The experiment was conducted as a randomized, placebo controlled, double blind, cross-over design. Subjects reported to the lab in a 12 hour fasted state and no food was consumed until all testing was completed. However, water was allowed ad libitum, and was measured and matched for both days of testing (mean intake for men = 1360 mL; mean intake for women = 691 mL).

As stated above, subjects reported to the lab in a fasted state (12 hours), without caffeine consumption during the past 12 hours. All testing was started by 0800 hours and the time for each subject was matched for both visits. Subjects were instructed not to exercise for the 48 hours prior to each testing day. Women reported during the first 7-9 days of their menstrual cycle in order to avoid any potential influence of estradiol on our blood markers. Upon arrival to the lab, subjects rested quietly for 30 minutes in a seated position. Following this quiet rest period, heart rate (via monitor) and blood pressure (via auscultation) were measured and a blood sample was obtained (Pre). Subjects were then provided their assigned condition (supplement or placebo) and ingested this in the presence of an investigator. Three additional blood samples were obtained from subjects during each visit, as follows: The second blood sample was collected 2 hours post intake, during which time subjects rested quietly (2 hr). Subjects then exercised on a treadmill for 30 minutes at an intensity equal to 65% of maximal heart rate reserve (A familiarization session was provided for this exercise bout during the initial screening visit). A third blood sample was taken within one minute of the cessation of exercise (2.5 hr). Subjects then rested quietly and a fourth blood sample was taken 90 minutes following the cessation of exercise (4 hr). Other than for the 30 minute exercise bout, subjects remained inactive in the laboratory during the entire 4.5 hour test period. Heart rate (HR), systolic (SBP), and diastolic (DBP) blood pressure were measured prior to each blood draw, and also at 1 hr and 3 hr post intake.

In addition to biochemical measures as described below, breath samples were collected from subjects during five-minute periods at all blood collection times, in addition to other times periodically during the four hour post ingestion measurement period. This was done in an attempt to determine if isolated breath sample collection, as opposed to continuous breath collection, could prove to be a useful methodology for assessing metabolic rate, kilocalorie expenditure, and substrate utilization measured via respiratory exchange ratio (RER) following capsaicinoid intake. The measurements were performed using indirect calorimetry via breath-by-breath collection (SensorMedics Vmax 229 metabolic system; Yorba Linda, CA). All gas collection took place in a temperature and humidity controlled laboratory, and both the flow sensor and gas analyzers were calibrated prior to data collection. Total oxygen consumption (L·min^-1^) was determined and total kilocalorie expenditure was estimated from this value. The RER was determined from gas collection (CO_2_/O_2_), and used as a crude measure of substrate utilization. Our results for these measures indicated that a sporadic collection period (i.e., 5-minute intervals) did not capture all potential changes in the above mentioned gas variables. Hence, these data are not presented within this manuscript. Future studies involving capsaicinoids should continue to use a continuous method of breath sample collection.

### Blood Collection and Biochemistry

A total of four venous blood samples (7 mL per draw) were taken from subjects' forearm via needle and Vacutainer^®^. Blood was immediately processed in a refrigerated centrifuge to obtain plasma (4°C for 15 min at 2000×g). Plasma samples were then stored in multiple aliquots at -80°C. All assays were performed on first thaw within four weeks of sample collection. Norepinephrine and EPI were determined using an enzyme linked immunosorbent assay (2-CAT ELISA, BA 10-1500; Rocky Mountain Diagnostics) following the instructions of the manufacturer (Labor Diagnostika Nord GmbH & Co. KG). In this competitive ELISA, NE and EPI are extracted by using a cis-diol-specific affinity gel, acylated, and then derivitized enzymatically. The coefficient of variation (CV) for NE and EPI in our lab is < 9% and < 8%, respectively. Free fatty acids were determined using the Free Fatty Acid Quantification Kit (K612-100) following the instructions of the manufacturer (BioVision). The CV for FFA in our lab is < 7%. Glycerol was determined using the Free Glycerol Determination Kit (FG0100) and Glycerol Standard (G7793), following the instructions of the manufacturer (Sigma Aldrich). The CV for glycerol in our lab is < 8%.

### Diet and Physical Activity

During the 24 hours before each test day, subjects consumed prepackaged meal replacement drinks (protein-rich ready-to-drink shake) and food bars. These contained a mix of protein, carbohydrate, and fat. Subjects were each provided with 3 shakes and 4 bars and instructed to consume as many of these as they desired, while consuming no other food or calorie containing drinks. The amount consumed during the day preceding the first test day was duplicated during the day preceding the second test day. The mean intake for men was 3 shakes and 4 bars, while for women this was 2 shakes and 2.5 bars. These amounts provided approximately 2000 kilocalories to men and 1250 kilocalories to women.

### Statistical Analysis

Biochemical data were analyzed using a 2 (condition) × 4 (time) analysis of variance (ANOVA). ANOVA was also performed for change from baseline (Pre) for all bloodborne variables. Condition and time were treated as fixed factors and subjects nested within sequence were treated as a random factor. Heart rate and blood pressure data were analyzed using a 2 (condition) × 6 (time) ANOVA. Statistical significance was set at p ≤ 0.05.

## Results

All subjects successfully completed both test days. The capsaicinoid supplement provided at the stated dosage was well tolerated, with no observed side effects related to gastric upset or discomfort.

### Hemodynamic Data

No condition x time interaction or condition effect was noted for HR, SBP, or DBP (p > 0.05). However, a time effect was noted for all variables (p < 0.05), with HR and SBP higher at 2.5 hr compared to Pre (due to the acute exercise bout) and DBP lower at 2.5 hr compared to Pre (due to the acute exercise bout). Hemodynamic data are presented in Table 2.

**Table 2:** Hemodynamic data for healthy adults consuming Capsimax™ and placebo in a randomized cross-over design

Variable	Pre	1 hr	2 hr (pre-ex)	2.5 hr (1 min post ex)	3 hr (30 min post ex)	4 hr (90 min post ex)
HR (bpm) *Capsimax™*	58 ± 12	57 ± 11	58 ± 11	115 ± 17*	73 ± 14	65 ± 15
HR (bpm) *Placebo*	60 ± 12	57 ± 11	58 ± 13	114 ± 14*	73 ± 14	65 ± 13
SBP (mmHg) *Capsimax™*	112 ± 8	114 ± 8	115 ± 11	140 ± 16*	115 ± 8	113 ± 12
SBP (mmHg) *Placebo*	112 ± 11	114 ± 11	116 ± 10	141 ± 16*	112 ± 11	111 ± 9
DBP (mmHg) *Capsimax™*	72 ± 7	72 ± 6	72 ± 7	64 ± 10*	67 ± 7	70 ± 8
DBP (mmHg) *Placebo*	71 ± 10	70 ± 7	70 ± 9	64 ± 10*	66 ± 8	68 ± 8

Data are Mean ± SD

No condition x time interaction or condition effect was noted for HR, SBP, or DBP (p > 0.05).

*A time effect was noted for all variables (p < 0.05); HR and SBP higher than Pre at 2.5 hr; DBP lower than Pre at 2.5 hr.

HR-heart rate; SBP-systolic blood pressure; DBP-diastolic blood pressure

### Biochemical Data

No interaction or condition effects were noted for EPI, NE, FFA, or glycerol (p > 0.05). However, a time effect was noted for all variables (p < 0.0001), with values higher than Pre at 2.5 hr for EPI and glycerol, at 2 hr and 2.5 hours for FFA, and at 2 hr, 2.5 hr, and 4 hr for NE (p < 0.05). Data for EPI and NE are presented in Table 3 and data for FFA and glycerol are presented in Table 4. When investigating the percent change data, glycerol for Capsimax™ was significantly higher than for placebo at 4 hr (p = 0.011). Also, FFA for Capsimax™ was significantly higher than for placebo at 2 hr (p = 0.025) and at 2.5 hr (p = 0.015). Data are presented in Table 5.

**Table 3:** Blood catecholamine data for healthy adults consuming Capsimax™ and placebo in a randomized cross-over design

Variable	Pre	2 hr (pre-ex)	2.5 hr (1 min post ex)	4 hr (90 min post ex)
Epinephrine (pg·mL^-1^) *Capsimax™*	41.54 ± 33.56	40.09 ± 29.83	69.34 ± 43.75 *	45.36 ± 39.36
Epinephrine (pg·mL^-1^) *Placebo*	37.65 ± 27.16	36.54 ± 21.31	65.08 ± 51.10 *	34.49 ± 22.12
Norepinephrine (pg·mL^-1^) *Capsimax™*	264.90 ± 132.66	378.63 ± 210.31**	491.08 ± 211.54**	440.10 ± 220.34 **
Norepinephrine (pg·mL^-1^) *Placebo*	242.03 ± 157.63	326.84 ± 185.89**	553.53 ± 200.58**	344.61 ± 210.45**

Data are Mean ± SD

No interaction or condition effects were noted for EPI or NE (p > 0.05).

* A time effect was noted for EPI (p < 0.0001); values higher than Pre at 2.5 hr.

** A time effect was noted for NE (p < 0.0001); values higher than Pre at 2 hr, 2.5 hr, and 4 hr.

**Table 4:** Blood free fatty acid and glycerol data for healthy adults consuming Capsimax™ and placebo in a randomized cross-over design

Variable	Pre	2 hr (pre-ex)	2.5 hr (1 min post ex)	4 hr (90 min post ex)
Free fatty acid (mmol·L^-1^) *Capsimax™*	0.19 ± 0.04	0.36 ± 0.19 *	0.59 ± 0.31 *	0.37 ± 0.27
Free fatty acid (mmol·L^-1^) *Placebo*	0.25 ± 0.10	0.30 ± 0.18 *	0.56 ± 0.31 *	0.33 ± 0.15
Glycerol (μg·mL^-1^) *Capsimax™*	9.46 ± 1.47	10.42 ± 2.94	28.73 ± 11.45 **	13.89 ± 8.90
Glycerol (μg·mL^-1^) *Placebo*	10.19 ± 4.79	10.73 ± 4.66	25.94 ± 8.02 **	10.53 ± 3.76

Data are Mean ± SD

No interaction or condition effects were noted for FFA or glycerol (p > 0.05).

* A time effect was noted for FFA (p < 0.0001); values higher than Pre at 2 hr and 2.5 hr.

** A time effect was noted for glycerol (p < 0.0001); values higher than Pre at 2.5 hr.

**Table 5:** Comparisons of percent change from Pre data between Capsimax™ and placebo for blood catecholamines, free fatty acids, and glycerol

Variable	Time	P value
Epinephrine	2 hr	0.69
	2.5 hr	0.95
	4 hr	0.81

Norepinephrine	2 hr	0.78
	2.5 hr	0.11
	4 hr	0.40

Free Fatty Acid	2 hr*	0.03
	2.5 hr*	0.02
	4 hr	0.28

Glycerol	2 hr	0.95
	2.5 hr	0.12
	4 hr*	0.01

* Significantly higher for Capsimax™ compared to placebo.

## Discussion

Our data indicate that ingestion of low dose capsaicinoid supplement can increase plasma FFA (2 hr and 2.5 hr) and glycerol (4 hr) concentrations compared to a placebo in a sample of healthy adults. There was no statistically significant effect of the capsaicinoid supplement on EPI or NE concentration compared to placebo. Finally, no statistically significant increase in HR, SBP, or DBP was noted with capsaicinoid supplementation (Table 2).

Several prior studies using human subjects have measured lipolysis using breath sample analysis rather than actual blood markers of triglyceride degradation. This former measure of lipolysis includes breath gas sample analysis using indirect calorimetry [23], or more specifically, a measure of the respiratory exchange ratio (VCO_2_/VO_2_). While this is a widely used tool in the study of metabolic rate and caloric expenditure, we wanted to include a more specific approach by including the measurement of blood FFA and glycerol concentrations. While many previous studies have used dosages of capsaicinoids ranging from 3 mg [16] to over 150 mg [24] while noting mixed findings for increased lipolysis, our data support the use of a very low dosage of capsaicinoids (2 mg) to increase FFA and glycerol concentrations. It is possible that a higher dosage of our specific capsaicinoid supplement would promote more dramatic effects. It is also possible, based on dissolution studies of the Capsimax™, that a longer time course of measurement may have been associated with more profound effects. Further research is necessary to test these hypotheses.

Previous studies have noted an increase in lipolysis following capsaicinoid intake [14,15], suggesting that this agent may aid in body weight/fat loss. However, regardless of the acute effects of capsaicinoids in terms of the lipolytic effects, the more important question pertains to the long-term effect of capsaicinoid condition on body weight/fat loss. A few studies have addressed this issue, noting greater fat oxidation during a 3-month weight maintenance phase (preceded by a 4-week very low calorie diet) when subjects received 135 mg capsaicin/day [15]. Despite this finding, no difference in weight regain was noted between capsaicin and placebo conditions. Snitker and colleagues had subjects ingest 6 mg/day of *non-pungent *capsinoids or a placebo for a period of 12 weeks and noted minimal difference between conditions in weight loss, body fat loss (statistically significant but of little physiological relevance), and fat oxidation [25]. In opposition to these reports, a significant reduction in visceral fat weight has been noted when capsaicin containing diets were fed to rodents [26]. Taken together, it is difficult to state with confidence the physiological meaning behind our noted increase in FFA (and to a lesser extent glycerol) as related to weight/fat loss over time. It is certainly possible that such an increase in lipolysis may be maintained following chronic intake of capsaicinoid supplementation, and be persistent for several hours throughout the day. If so, it can be hypothesized that such supplementation may indeed result in a significant effect on body weight/fat loss over time. Of course, future investigations are needed to support these hypotheses.

Finally, our data corroborate previous findings of increased fatty acid release in healthy adults [27-29], as indicated by our 2 and 2.5 hour sample analysis (Table 4). Our objective in the present study was to investigate the effect of a standard dosage of capsaicinoid (that typically consumed within an over-the-counter dietary supplement) in healthy adults, regardless of body weight. However, future studies may provide a dosage based on kg body weight rather than a standard dosage, in an attempt to more specifically delineate potential differences in the physiological effects of capsaicinoid supplementation in adult subjects.

In conclusion, we report that a low dose capsaicinoid supplement can increase plasma FFA concentrations and glycerol compared to a placebo. This finding is observed at 2 to 2.5 hr for FFA and 4 hr for glycerol post ingestion of the supplement. The supplement had no statistically significant effect on blood EPI or NE concentration, or on HR, SBP, or DBP. Finally, these data support the well-documented finding of increased fatty acid release and glycerol in response to an acute exercise bout. Further study is needed to determine if more pronounced effects of capsaicinoid intake can be observed using a longer time course of measurement and/or a higher dosage of treatment, with and without acute exercise. More importantly, the chronic effects of Capsimax™ on weight/fat loss require investigation.

## Competing interests

Financial support for this work was provided in part by OmniActive Health Technologies. The authors have no financial interest in this company.

## Authors' contributions

RJB was responsible for the study design, biochemical work, and manuscript preparation; REC was responsible for data collection, blood collection and processing, and assistance with manuscript preparation; S Shastri was responsible for assistance with the study design and manuscript preparation; S Suvarnapathki was responsible for the statistical analyses. All authors read and approved of the manuscript.

## Location of work

Cardiorespiratory/Metabolic Laboratory, Department of Health and Sport Sciences, University of Memphis, Memphis, TN, USA
